# What Does Plant-Based Vaccine Technology Offer to the Fight against COVID-19?

**DOI:** 10.3390/vaccines8020183

**Published:** 2020-04-14

**Authors:** Sergio Rosales-Mendoza, Verónica A. Márquez-Escobar, Omar González-Ortega, Ricardo Nieto-Gómez, Jaime I. Arévalo-Villalobos

**Affiliations:** 1 Facultad de Ciencias Químicas, Universidad Autónoma de San Luis Potosí, Av. Dr. Manuel Nava 6, San Luis Potosí 78210, Mexico; vero_marquez_333@hotmail.com (V.A.M.-E.); omar.gonzalez@uaslp.mx (O.G.-O.); ricardoxvi@hotmail.com (R.N.-G.); aviivan_jaime@hotmail.com (J.I.A.-V.); 2 Sección de Biotecnología, Centro de Investigación en Ciencias de la Salud y Biomedicina, Universidad Autónoma de San Luis Potosí, Av. Sierra Leona 550, Lomas 2ª Sección, San Luis Potosí 78210, Mexico

**Keywords:** zoonosis, multiepitope vaccine, molecular farming, oral vaccines, epitope-based vaccine, mucosal immunization

## Abstract

The emergence of new pathogenic viral strains is a constant threat to global health, with the new coronavirus strain COVID-19 as the latest example. COVID-19, caused by the SARS-CoV-2 virus has quickly spread around the globe. This pandemic demands rapid development of drugs and vaccines. Plant-based vaccines are a technology with proven viability, which have led to promising results for candidates evaluated at the clinical level, meaning this technology could contribute towards the fight against COVID-19. Herein, a perspective in how plant-based vaccines can be developed against COVID-19 is presented. Injectable vaccines could be generated by using transient expression systems, which offer the highest protein yields and are already adopted at the industrial level to produce VLPs-vaccines and other biopharmaceuticals under GMPC-processes. Stably-transformed plants are another option, but this approach requires more time for the development of antigen-producing lines. Nonetheless, this approach offers the possibility of developing oral vaccines in which the plant cell could act as the antigen delivery agent. Therefore, this is the most attractive approach in terms of cost, easy delivery, and mucosal immunity induction. The development of multiepitope, rationally-designed vaccines is also discussed regarding the experience gained in expression of chimeric immunogenic proteins in plant systems.

## 1. Introduction 

The coronaviruses (CoVs) are enveloped viruses having a positive-sense, single-stranded genomic RNA [1] and are grouped into four genera: α-CoVs, β-CoVs, γ-CoVs, and δ-CoVs. The ones that affect mammals are α- and β-CoVs, while the other two genera infect birds and could also infect mammals [2]. Lately, the coronavirus has become a remarkable concern for global health after the diagnosis of a cluster of unknown pneumonia patients in December 2019 in Wuhan, China. The outbreak was associated with workers in the Wuhan Wholesale Seafood Market, in which live exotic animals are sold [3]. The pathogen was named SARS-CoV-2 given its similarity (70% of genomic similarity) to SARS-CoV-1, which was responsible for the 2002–2003 severe acute respiratory syndrome epidemic (SARS). Until now, the official and accepted name for this new coronavirus strain is COVID-19 virus, an acronym for coronavirus infectious disease 2019 [1]. A systematic genomic analysis has revealed 380 amino acid substitutions between SARS-CoV-1 and SARS-CoV-2, which is a starting point to study its functional and pathogenic divergence [4].

Since the end of 2019, COVID-19 has disseminated over the world. Every continent has reported presence of the virus and the World Health Organization has declared a Public Health Emergency of International Concern on 30 January, 2020 [1]. On 11 March, 2020, pandemic status was announced. This is the first pandemic caused by a coronavirus species. The symptoms associated with COVID-19 comprise flu-like symptoms including fever, cough, dyspnea, myalgia, and asthenia. SARS-CoV-2 can cause pneumonia, acute respiratory distress syndrome, sepsis, and septic shock that can lead to death [5]. COVID-19 patients usually exhibit fever and lower respiratory tract symptoms and the estimated incubation time for the disease is 14 days. The most accepted COVID-19 diagnosis method is real-time PCR to detect its genomic RNA, which was implemented thanks to the rapid sequencing and availability of the genomic sequence. The processing of respiratory or blood samples allows detection in a few hours [2]. At the end of March, the global number of confirmed cases of COVID-19 was around 441,000 which has led to approximately 20,000 deaths in 172 countries/regions [6]. China has the majority of the cases with more than 81,000 cases recorded, followed by Italy with more than 69,000 recorded cases [1].

Health control measures mainly include drugs to relieve symptoms and maintain vital functions. The most frequent form of human–human transmission is by spread through aerial drops that are discharged upon sneezing, coughing, or exhaling [7]. Additionally, transmission by contact is also prominent especially through the oral, nasal, and ocular mucous membranes [8]. Therefore, the main immediate measures to prevent transmission include hand washing, wearing masks, and following good hygiene practices when coughing and sneezing [9].

## 2. Development of Therapeutic and Prophylactic Treatments against COVID-19

Presently, there is no specific treatment for COVID-19. As an emerging pathogen, several knowledge gaps exist about the SARS-CoV-2 virus and the coming months will be critical to refine clinical management and advancement in the validation of possible treatments. A myriad of efforts is ongoing to perform clinical trials and determine the efficacy of preexisting drugs, with hydroxychloroquine and Remdesivir as the most promising candidates [10]. Receptor blockers are also under evaluation [11], along with the assessment and development of monoclonal antibodies. Moreover, transfusions using plasma from individuals recovered from SARS-CoV-2 infection (containing neutralizing antibodies) are under exploration with promising findings [12]. Nonetheless, vaccination is the most effective approach to control and ultimately eradicate infectious diseases. Since SARS-CoV-2 possesses high transmissibility (asymptomatic and presymptomatic virus shedding, which results in a high number of patients with mild symptoms), the development of vaccines is an urgent goal to fight against this pathogen. The straightforward path to generate vaccine candidates is the technology of inactivated vaccines, which can be formulated with SARS-CoV-2 virions previously inactivated by chemical or physical treatments. A vaccine based on live-attenuated virus is another possible approach [13]. For SARS-CoV-1 and MERS-CoV, inactivated vaccine candidates have induced robust humoral responses leading to virus neutralization. Alternatively, the construction of a chimeric attenuated virus constitutes an interesting path. For instance, the influenza virus can be used as a scaffold to expose SARS-CoV-2 antigens and generate a bivalent vaccine targeting two relevant pathogens causing respiratory diseases [14]. Similarly, adenoviral vectors could be applied in this field [15]. Vaccines based on whole viruses are associated with concerns about antibody-dependent enhancement of viral infection, reactogenicity, and strain reversion to pathogenic forms, among other safety issues [16]. Therefore, the path for vaccine development will require a detailed characterization, not only in terms of efficacy but also safety [17].

**Figure 1:**
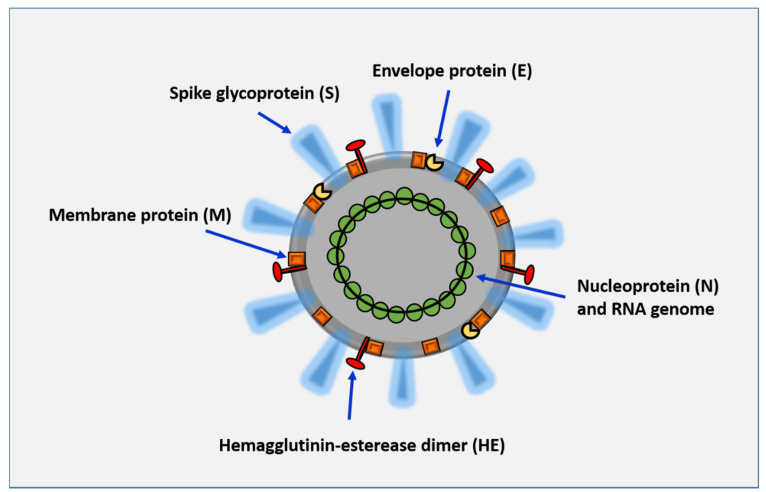
Structure of the SARS-CoV-2 virus. The virus is formed by an envelope membrane associated with the following structural proteins: spike protein (S), which mediates binding to the host cell receptors and considered a critical target for the induction of antibodies capable of neutralizing the virus; hemagglutinin-esterase dimer (HE), which acts as a potent mediator of attachment and destruction of sialic acid receptors on the host cell surface; a membrane glycoprotein (M), which is important to generate the virus; and the envelope protein (E), which adheres to the M protein to form the viral envelope. The viral structure also comprises a nucleocapsid protein (N) that, along with the RNA genome, produces the nucleocapsid.

Although inactivated vaccines will perhaps be the main avenue for generating the first experimental vaccines, alternative approaches should be explored in parallel, namely the development of subunit vaccines. Since the coronavirus spike (S) glycoproteins initiate entry into cells; they are considered the primary target for neutralizing antibodies (Figure 1). Cytotoxic T-lymphocyte (CTL) responses are also of key relevance to protect against viral infections [18]. Under these principles, vaccine development against COVID-19 has been initiated and efforts announced in this field include the development of RNA-based vaccines by CureVac [19,20], and an RNA vaccine candidate formulated with a novel lipid nanoparticle (LNP) carrying mRNA encoding for a full-length, prefusion stabilized S protein [21]. Another candidate (developed by Shenzhen Geno-Immune Medical Institute [22]) consists of a multiepitopic vaccine based on the generation of artificial antigen presenting cells through transduction as a way to express viral antigens and immune modulatory genes to ultimately activate T cells. 

Although the conventional developmental paths for vaccines take several years, the vaccine against hand, foot, and mouth disease (HFMD) that was approved in China; demonstrates how joint efforts can lead to new vaccines that are accepted in relatively short periods after a pathogen emerges [23]. 

Similarities with the SARS-CoV-1 virus constitute a valuable reference for vaccine development, however it should be considered that the genetic variability of SARS-CoV-1 seems higher as more than two variants have been described. Despite this, the cross protection of SARS-CoV-1 and MERS experimental vaccines remains to be explored. Thus far, there is evidence that some SARS-CoV-1 neutralizing antibodies cross react with SARS-CoV-2, however extensive research in this regard is needed. The epitopes conserved among SARS-CoV-1 and SARS-CoV-2 have been identified and proposed for vaccine development [24]. 

The race for generating fundamental knowledge on COVID-19 is leading to rapid advances, which will be useful to refine vaccine design. For instance, Walls et al. [25], reported cryo-EM structures of the ectodomain trimer, which will be of critical importance for designing vaccines and viral entry blockers. They also found evidence of potent inhibition of SARS-CoV-2 entry into host cells by using murine polyclonal antibodies against SARS-CoV-1, which suggests that conserved S epitopes among these viruses are relevant for immunization and perhaps preexisting immunization models will be useful in the fight against COVID-19. The characterization of other key targets for drug design has been accelerated (e.g., the protease X-ray structure has been recently described [26]). Therefore, these recent advances will set the basis to accelerate the development of COVID-19 subunit vaccines taking into consideration the SARS-CoV-1 vaccine development, especially the approach targeting the S protein. 

In parallel with these advances in vaccine design, defining vaccine platforms amenable for rapid and large scale production is required. These must keep in mind that low cost, safe, and easy distribution and delivery are the required attributes to implement broad coverage of vaccination campaigns; especially in the developing countries.

## 3. Molecular Farming and the Plant-based Vaccines Technology

Genetically engineered plants constitute a consolidated platform for the manufacturing of biopharmaceuticals. Plants have been used over the last three decades for this purpose. Thus far, a diverse group of biopharmaceuticals has been functionally produced in plant systems that include antibodies, vaccines, growth factors, and cytokines [27]. Remarkably, a recombinant enzyme produced in carrot cells has been already approved by the FDA for Gaucher’s disease treatment [28]. The main advantages of plant-based platforms include the inability to replicate human pathogens (diminishing the risk of contamination and making the purification process less strident), use of unsophisticated bioreactors, and efficient synthesis of complex proteins (multimeric or glycosylated) [29,30]. 

### 3.1. Approaches for Recombinant Protein Expression in Plants

The current expression approaches for recombinant proteins using the plant cell as host comprise stably-transformed plants at the nuclear or chloroplast levels and transiently-transformed plants. These expression modalities are summarized in Table 1. The conventional approach for expression of transgenes in plants comprises transgene insertion into the nuclear genome. Currently, *Agrobacterium*-mediated transformation is the most popular method to achieve this modification since this bacterium has the ability to transfer large segments of DNA with minimal rearrangement at high efficiency with low number of insertions. Nonetheless, the transgene is randomly inserted into the genome, which often leads to positional effects that make expression levels unpredictable and interruption of endogenous genes a possibility. Another limitation is the induction of silencing mechanisms that hamper productivity. Nevertheless, it should be considered that new technologies are emerging to cope with these limitations by providing ways to achieve site-directed insertion through a number of mechanisms [31]. 

In the case of transplastomic technologies, they typically comprise the site-directed insertion of the foreign DNA into the chloroplast genome in an event induced by homologous recombination. The main advantage of this technology is the high protein yield, which is a consequence of the high copy number of the transgene and the fact that silencing events have not been reported thus far, and position effects are avoided due to the site-directed insertion. In addition, transgene confinement is achieved given the maternal inheritance shown by most plant species and several proteins can be produced from a single transformation event through the use of operon-like arrangements [37,38]. For more information regarding transplastomic technologies, readers are referred to reviews published by [36,39].

Another approach to express heterologous protein in plants relies on the use of viral-based vectors, which exploit the efficient promoters, UTRs, and DNA/RNA replication mechanisms found in plant viruses. Interestingly, the *Agrobacterium*-mediated delivery of viral vectors has consolidated as a highly efficient strategy to achieve rapid production of biopharmaceuticals with yield up to 5 g of protein per kg of fresh plant biomass. The tobamoviruses, potexviruses, tobraviruses, geminiviruses, and comoviruses have served as a basis for the development of transient, efficient expression systems in plants [40]. This technology, however, demands the purification of the target protein given the presence of toxic compounds (e.g., alkaloids) in the typical host (*Nicotiana benthamiana*), as well as bacterial residues, especially endotoxins. Therefore, at present, this technology is applied for the formulation of injectable or nasal vaccines.

### 3.2. Current Scenario of Plant-Based Vaccines 

Thus far, some plant-vaccine candidates have entered clinical trials, including candidates against swine influenza, rabies, and hepatitis B [41]. The most promising candidates are the influenza vaccines developed by Medicago Inc. that rely on using a non-replicative vector carrying viral regulatory sequences to mediate the transient expression of Hemagglutinin (HA) in *N. benthamiana*, which has led to injectable vaccine candidates [42,43]. Overall, these vaccines have been seen as safe and their immunogenic properties have been positively evaluated using in vitro assays with human and mice cells. The trials conducted in volunteers revealed proper immunogenicity with no serious adverse effects (see Table 2). Moreover, a group of respiratory diseases has been targeted through the plant-based production of antigens with promising immunogenicity in preclinical trials [44]. 

Nonetheless, the ultimate goal for low-cost vaccine development could be achieved by generating oral formulations not requiring purification and being composed of freeze-dried biomass encapsulated in gelatin pills or tablets. Under this focus, the goal is to trigger specific immune responses via the gut-associated lymphoid tissues (GALT). For this purpose, edible plants lacking toxic metabolites should be used. Perhaps the main disadvantage of this technology is the long time required to generate transformed lines of edible crops efficiently expressing the antigen of interest (e.g., rice or corn transgenic lines or transplastomic lines) [51]. These oral vaccines will overcome the disadvantages of injectable vaccines such as painful administration and the risk associated with invasive delivery routes. In terms of costs, avoiding the requirements of sterile devices and trained personnel represent substantial savings. The fact that plant-based vaccine formulations will not require antigen purification will be, without a doubt, the main factor that will make them low-cost alternatives [52], which is necessary to provide wide vaccination coverage in developing and low-income countries. Recent promising reports in the viability of formulating oral vaccines include the case of the Hepatitis B virus surface antigen [53] and models of immunotherapy against allergy with promising results [54], among other diseases. 

Therefore plant-based vaccines are becoming a reality, although it should be recognized that the progress has been slower than initially expected. This is particularly true for the development of oral vaccines, with the main drawbacks being poor characterization of antigen stability, bioavailability, and reproducibility [51,55]. 

**Figure 2:**
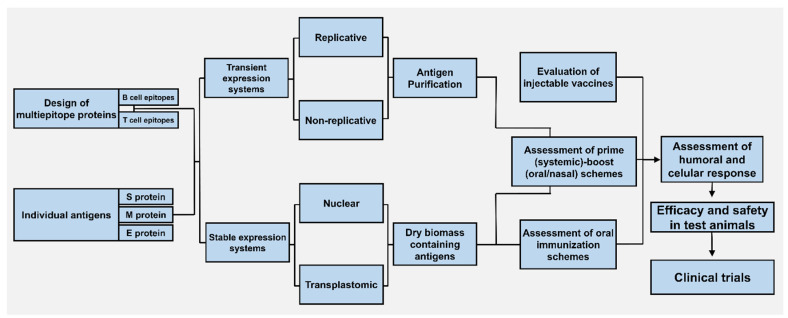
Developmental paths for plant-based vaccines. Vaccine antigens can comprise full length viral proteins or chimeric proteins rationally designed to serve as multiepitopic vaccines. This is based on the selection of T cell and B cell epitopes with the aid of immune bioinformatics tools and experimental data of their protective capacity. Current genetic engineering technologies allow expressing target antigens either stably or transiently, which allow exploring distinct immunization approaches. Transient expression typically requires purification of the antigen—which is useful for the formulation of injectable vaccines—whereas stable transformation, especially in edible crops, allows implementing oral immunization schemes without purification. Combined schemes, using pure antigens for priming by parenteral administration and plant biomass containing the antigen for oral boosting, are likely ideal approaches capable of inducing long-term protection in several compartments, especially in mucous membranes.

The currently available expression strategies for foreign proteins in plants allow projecting the generation of several vaccine candidates against COVID-19 in the short term. The expression approach and targeted organelle will depend on the nature of the selected target antigen. In the following section, possible avenues for the development of plant-based vaccines are envisaged (Table 3 and Figure 2).

## 4. Possibilities to Develop Anti-COVID-19 Plant-based Vaccines

### 4.1. Virus-Like Particles (VLPs)

One prominent approach for vaccine design is based on the use of virus-like particles (VLPs), which are macromolecular complexes resembling viruses, but lacking their genome. In this way, VLPs mimic the native structure of viruses, but are not infective. This avoids the disadvantages of vaccines formulated with attenuated or inactivated viruses that include reactogenicity and reversion to pathogenic forms [61,62]. A myriad of reports on the production of VLPs can be found in the literature that comprise the cases of the influenza virus, human papillomavirus, human immunodeficiency virus, foot and mouth disease virus, Norwalk virus, rift valley fever virus, and hepatitis B virus [61,62,63,64]. 

The precedents of SARS-CoV-1 and MERS antigens expressed in recombinant systems leading to the formation of VLPs constitute important guides for the topic of COVID-19 vaccine development. Mortola and Roy (2004) produced coronavirus VLPs applying baculovirus/insect cells expression (*Sf9* cells), which was based on the following structural proteins: S (spike), E (envelope), M (membrane), and N (nucleocapsid) of SARS-CoV, either individually or simultaneously. Simultaneous expression at high levels was achieved for S, E, and M proteins, leading to an efficient VLPs assembly and release, evidenced by electron microscopy and immunofluorescence. The authors demonstrated that the formed VLPs were similar in morphology to the SARS-CoV-1 virions [65]. Another group reported in the same year that M and E proteins were sufficient for the efficient formation of VLPs in insect cells [66].

In 2007, the immunogenicity of SARS-CoV-1 VLPs was first described by evaluating insect cell-made VLPs based on the M, E, and S proteins. Electron microscopy demonstrated VLPs formation in co-infected insect cells. Mice subjected to an immunization scheme consisting of four subcutaneous (s.c.) doses of VLPs emulsified with Freund’s adjuvant showed high antibody titers against SARS CoV. Furthermore, VLPs elicited cellular immunity following increased production of IFN-γ and IL-4 [67]. Subsequent in vitro assays using VLPs designed with the bat-isolated coronavirus protein S and the SARS-CoV-1 proteins E and M; demonstrated ability to stimulate DCs in terms of cytokine induction, evidenced by IL-6 and TNF-alpha production. Furthermore, the study indicated that IFN-γ+ and IL-4+ CD4+ T cells increased in co-culture with DCs pre-exposed to the VLPs tested [68].

Given that immunization by mucosal routes is the most pertinent for vaccination, SARS-CoV-1 VLPs were analyzed in a mouse model based on intraperitoneal or nasal immunization. Both routes led to SARS-CoV-1-specific IgG responses, IgG levels in the groups immunized intraperitoneally were higher. Nasal immunization usually results in the induction of secretory IgA responses at the genital tract, saliva, and lungs; a type of response that is not efficiently induced by systemic administration. In the mentioned study, IgA production at the intestinal tract was higher for intraperitoneal administration when compared to intranasal administration, however only genital tract secretions from the i.n.-immunized group showed neutralization activity [69].

Another approach for the production of VLPs consisted in co-expressing the SARS-CoV-1 S protein and the M1 influenza protein in the baculovirus/insect cell expression system. Interestingly, the chimeric VLPs showed similar size and morphology compared to the wild type SARS-CoV-1. Immunogenicity and protective efficacy of these chimeric VLPs were tested in a mouse lethal challenge model. Complete protection from death was observed in mice vaccinated intramuscularly or intranasally with the chimeric VLPs, which contained 0.8 µg of the SARS-CoV-1 protein S [70].

In the case of the Middle East respiratory syndrome (MERS), VLPs with proteins S, E, and M were generated using the baculovirus/insect cell expression system by Wang et al. (2017) [71]. Electron microscopy and immunoelectron microscopy revealed high structural similarity between the MERS-CoV VLPs and the native virus. Rhesus macaques inoculated with MERS-CoV VLPs and aluminum adjuvant showed final virus neutralizing antibody titers reaching 1:1280; inducing T-helper 1 (Th1) cell-mediated immunity.

In a more recent study, the structural proteins of MERS-CoV were expressed in silkworm larvae and Bm5 cells for the development of vaccine candidates. However, protein S was not detected in the form of VLPs despite the fact that E and M proteins were secreted to the culture supernatant. Surfactant treatment and mechanical extrusion using protein S or Bm5 cells expressing structural proteins allowed producing nanovesicles exhibiting protein S with a diameter ranging from 100 to 200 nm as revealed by immuno-TEM [72].

Although insect cells possess a protein processing capacity similar to that of higher eukaryotic cells, the protein processing pathways are not totally equivalent [73]. In terms of N-acetylglycosylation, insect cells have the ability to assemble N-glycans to a nascent polypeptide. However, they have unusual final processing activity mediated by β-*N*-acetylglucosaminidase (soluble or membrane-bound form), which trims an intermediate product common to mammalian and insect pathways resulting in the insect-specific paucimannose as a final product that avoids the galactosylation process occurring in mammals [74].

Considering that VLPs of enveloped viruses have been successfully expressed in plants by expressing the HA protein, it is anticipated that SARS-CoV-2 VLPs could be assembled by expressing the S protein. In fact, although not published in the literature yet, Medicago has described the generation of VLPs in plants [75]. For this purpose, nuclear expression targeting the trans-Golgi secretion route is proposed to yield a protein subjected to the glycosylation and secretion machinery that could make possible the production of VLPs [76]. Therefore, adoption of these works would be valuable during the development of COVID-19 plant-based vaccines (see the section below).

Besides VLPs resembling the SARS-CoV-2 virus, another possible approach is to adopt SARS-CoV-2 epitopes and express them in chimeric VLPs. In this way, a VLP from an unrelated virus can serve as the scaffold to present the target SARS-CoV-2 epitopes. For this purpose, the hepatitis B core protein has been applied as scaffold to display unrelated antigens of some pathogens. In particular, this has been applied at the immunodominant c/e1 loop region, which allows displaying the target on the spike structures of the VLPs [77]. A recent review revealed that at least 97 vaccine candidates have been developed based on plant viruses covering infectious agents, cancer, and autoimmune disorders [78].

The glycosylation patterns of proteins that form VLPs can impact their immunogenicity and protective capacity. Interestingly, glycoengineering strategies have been successfully implemented for plants, which allows diversifying their application as hosts for the production of biopharmaceuticals. This is a relevant aspect considering that plants lack the ability to perform glycosylation, which is a characteristic of mammalian systems. For instance, plants perform beta1,2-xylosylation, core alpha1,3-fucosylation, and the addition of a second N-acetylglucosamine (GlcNAc) to the mannose core. Moreover, plant glycans lack β (1,4’)-galactose and sialic acid, as well as bi-antennary N-glycans [79]. In some cases, such as antibody production, these differences on glycosylation can be associated to adverse effects that include generation of immunogenic activity, which can eventually lead to blocking antibodies against the therapeutic antibody. Nonetheless, in the case of vaccines these differences could add more immunogenic potential and improve vaccine efficacy [80]. In any case, it is of interest to comment that glycoengineering has allowed developing knock down *N. benthamiana* plants for beta1,2-xylosyltransferase (XylT) and alpha1,3-fucosyltransferase (FucT) genes, which showed significant reduction in the production of xylosylated and/or core alpha1,3-fucosylated proteins with no phenotypic alterations [81]. These lines will be valuable tools to study the impact of glycosylation in the efficacy of COVID-19 VLPs, which will allow optimizing the plant-based vaccines.

### 4.2. Multiepitopic Vaccines 

Another approach that deserves attention is the development of multiepitopic vaccines, which offers the opportunity of achieving a fine rational vaccine design by selecting epitopes that potentially induce robust and protective immune responses. At the same time, this approach will discard those related to non-protective responses or even those inducing antibody-dependent enhancement of the disease. In this way, a highly efficacious and safe vaccine can be obtained. Of special interest are the reports proving that specific S protein epitopes enhance the disease upon a pathogen challenge, which highlights the relevance of recurring to the rational design of vaccines to ensure not only immunoprotection, but safety [82]. 

For the case of multiepitope vaccines against infectious agents, several reports have focused on selecting the most promising Th, B cell, and T cell epitopes that might allow the rational design of a vaccine inducing robust protective responses, while at the same time avoiding deleterious or non-relevant responses. One key factor that adds importance to the development of multiepitopic vaccines against viral disease is genetic variability. In fact, it has been suggested that the SARS-CoV-2 virus evolved into two major types, L and S. The L type (∼70%) predominates versus the S type (∼30%), with the latter being proposed as the ancestral version. The L type is more aggressive than the S type and the analysis performed by Tang et al. [83] suggests that human interference influenced the prevalence of L and S types right after the outbreak appearance. Another study has also suggested a rapid evolution of this coronavirus [84]. Therefore, the design of multiepitopic vaccines must adopt the selection of epitopes conserved among the viral variants with the capacity to induce neutralizing humoral responses as critical criterion. It should also be considered that CTL responses are relevant to cope with COVID-19 [85].

The selected epitopes are the targets to which adaptive immune responses should be induced, but they lack the required complexity to trigger robust immune responses. Therefore, a carrier is required to increase antigen complexity and, through adjuvant effects, enhance potency of the induced immune response, making it properly polarized. Among these carriers, the B subunit of the cholera toxin and the heat-labile enterotoxin from *Escherichia coli* have been extensively used to carry unrelated antigens. The main advantage of these molecules is their adjuvant effects at the mucosal levels, as well as their ability to efficiently deliver the antigens to the submucosa thanks to a mechanism of translocation mediated by binding to the GM1 receptor at the epithelial cells in the mucosal surfaces [86]. Once at the submucosa, the antigen is efficiently captured and processed by antigen presenting cells, which subsequently induce robust mucosal and systemic Th1 immune responses at the lymph nodes. The design of LTB/CTB-based chimeras carrying SARS-CoV-2 epitopes is an interesting approach that should be explored. Proline-containing linkers have been used to properly display unrelated antigens without altering the ability of those carriers to form the pentameric structure responsible for GM1 binding [57]. The accumulation of those chimeric proteins in the ER is advisable, although toxicity has been reported for some plants accumulating high levels of those recombinant proteins [87]. 

Given that plant systems have been successfully adopted for the production of multiepitope proteins, the generation of such vaccines against COVID-19 is considered highly viable [88,89,90]. Besides expressing chimeric proteins, multiple antigen expression can be achieved by transplastomic technologies (operon-like expression) or at the nuclear level using the picornaviral 2A sequence that allows releasing individual antigens from a single coding gene [91]. 

### 4.3. Immune Complexes

The production of immune complexes (ICs) in plants is another approach that renders highly immunogenic agents. ICs consist of antigens complexed to antibodies recognizing them, constituting macromolecular entities that are efficiently captured and processed by antigen presenting cells [92]. This results in the induction of robust humoral and cell-mediated immune responses [59,93]. Taking advantage of the machinery of plant cells for protein synthesis and processing, they have been exploited as antibodies and ICs factories [94]. For instance, ICs based on the tetanus toxin fragment C fused to a monoclonal antibody were produced in transgenic tobacco plants. These ICs were highly immunogenic, inducing immunoprotective effects when administered without accessory adjuvants by the s.c. (subcutaneous) route in mice [95]. This approach has also been applied to the case of the Ag85B and Acr antigens from *Mycobacterium tuberculosis* and the GP1 antigen from the Ebola virus [96,97]. Perhaps the main disadvantage of this approach is the requirement of defined antibodies targeting the antigen, which is not available yet for SARS-CoV-2. However, it should be considered that the cross reactivity of anti-SARS-CoV-1 S protein antibodies with the counterpart of SARS-CoV-2 has been reported [3].

### 4.4. Elastin-Like Polypeptide Fusions

The purification of antigens is a laborious and expensive activity as part of the production of injectable vaccines [97]. One alternative to simplify purification relies on the fusion of elastin-like polypeptides (ELP) that possess a unique property named reversible phase transition, which allows precipitating the protein of interest by changing the temperature. This approach is an alternative to the complex/expensive affinity chromatography [98] that has been applied to develop plant-based vaccines candidates with relevant findings. For instance, the *M. tuberculosis* antigens Ag85B and ESAT-6 were fused to ELP and expressed in transgenic tobacco [99], and the plant-made antigens were subcutaneously (s.c.)administered to mice and able to trigger long-lasting humoral immune responses. The ELP did not negatively impact the immunogenic properties of the antigens. The haemagglutinin (HA) antigen from the influenza virus has also been expressed under this modality in tobacco, where antigen purification was simplified and the obtained product induced neutralizing antibodies in mice after two s.c. immunizations. ELPylation did not alter the immunogenicity of HA [99]. Therefore, these precedents suggest that the ELP technology is a possible approach to be explored for the production of antigens against the SARS-CoV-2 virus.

The common steps to the above-mentioned antigen design will comprise determining antigen yields and antigenic activity, assessing the immunogenic activity in test animals under distinct administration routes, and validating the safety and stability of the vaccine. The latter will be critical to justify the application and get approval to conduct clinical trials. 

## 5. Efforts to Develop Plant-Based Vaccines against MERS and SARS-CoV-1

A key precedent for this field is the vaccine candidates already reported for SARS-CoV-1 and MERS, which are both closely related to SARS-CoV-2. Following nuclear expression approaches, the N-terminal fragment of the SARS-CoV-1 S protein (S1) was expressed in tomato and low-nicotine tobacco plants using an *Agrobacterium*-mediated method. Mice orally immunized with this transgenic tomato revealed significantly increased levels of SARS-CoV-1-specific IgA. Sera of mice parenterally primed with the transgenic tobacco showed the presence of anti-SARS-CoV-1-IgG [32].

Another study focused on expressing a chimeric protein of GFP and amino acids 1-658 of the SARS-CoV-1 spike protein (S1:GFP) by using tobacco leaves subjected to transient expression. After microscopy localization, the fusion protein was observed in the cytosol and the periphery of the nucleus. Stable transgenic tobacco and lettuce plants were generated by *Agrobacterium*-mediated transformation. The expression of the chimeric antigen was also achieved in tobacco chloroplasts. However, no immunization assays were performed [33,100].

The SARS-CoV-1 nucleocapsid (rN) protein of 423-aa was transiently expressed in *N. benthamiana*. Yields reached up to 79 µg/g of leaf fresh weight (corresponding to 0.8%–1% of the Total Soluble protein, TSP) at 3 dpi under silencing suppression conditions (p19). Mice immunization revealed that three doses led to effective B-cell maturation and differentiation, achieving high levels of IgG1 and IgG2a. IFN-γ and IL-10 were up-regulated in splenocytes, while the expression of IL-2 and IL-4 was not [33]. The nucleocapsid protein (N) and the membrane protein (M) were transiently expressed in *N. benthamiana*. Yields reached up to 3–4 µg/g fresh leaf weight (0.2% TSP) for the N protein, while the M protein yields were 0.1%–0.15% TSP. The purified N protein reacted with human sera having N-specific antibodies. No immunization assays were performed [35]. These studies constitute a valuable guide to select the most convenient expression platforms for specific SARS-CoV-2 antigens.

## 6. Relevance of Mucosal Vaccines and Prime-Boosting Immunization Schemes 

Although most of the vaccines against respiratory diseases are administered by parenteral routes, it should be recognized that immune profiles deserve improvements, especially in terms of immunogenicity and efficacy in the elderly [101]. Mucosal vaccines, especially intranasal-administered vaccines, are a promise for immunization against respiratory diseases given the protections induced in lungs and other mucosal tissues that are critical ports for pathogen entry. Critical alternatives for the development of mucosal vaccines include guaranteeing antigen delivery and immunostimulating capacity at the mucosa, while at the same time being minimally reactogenic/toxic. Among the immunopotentiator molecules that can be explored are bacterial toxin derivatives, toll-like receptor ligands, and cytokines [102,103].

Although some companies have already started the production of plant-based VLPs vaccines [75,104] using transient expression systems, these efforts are mainly directed to developing injectable vaccines. Therefore, it is imperative to start developing alternatives related to mucosal immunization. For this purpose, generating plants stably expressing SARS-CoV-2 antigens will open new avenues for the exploitation of this technology. This approach will result in the production of edible plant material containing SARS-CoV-2 antigens that could be used for the development of oral boosting agents. Interestingly, this concept has been assessed by some groups with relevant results. In the case of a vaccine targeting the hepatitis B virus, a scheme comprising intramuscular (i.m.) boosting with the pure HBsAg antigen followed by double oral boosting with plant material containing HBsAg at six week intervals led to a comparable response to that induced by the standard intramuscular (i.m.) vaccination scheme [53]. Moreover, in the case of poliomyelitis vaccination, a plant-based vaccine based on transplastomic plants expressing the VP1 antigen fused to a carrier has been assessed as oral boosting agents after priming with the inactivated polio vaccine administered subcutaneously. Boosting with the plant-made VP1-based antigen significantly enhanced VP1-IgG1 and VP1-IgA titers when compared to the response in animals not receiving boosts. The scheme also allowed inducing neutralizing antibodies for the three poliovirus Sabin serotypes when two doses of IPV and plant-cell oral boosts were administered, whereas a single dose of IPV resulted in low neutralization potential [105]. In this way, freeze-dried plant material can be used as a low-cost vaccine rendering thermostability and easy to administer features. 

Similarly, pure antigens produced in plants have been assessed with promising results in schemes where a conventional vaccine is used as a priming agent and the plant-made antigen is administered by the oral route as boosting [106]. Exploring these schemes will be useful to achieve the best immune profile against SARS-CoV-2 in terms of safe and long-lasting protective immune responses. In fact, the potential of using prime-boosting schemes through different immunization routes has been proven as an efficacious approach to achieve the desired immune profiles [107]. 

## 7. Conclusions

The emergence of COVID-19 has led to a global emergency that demands the development of new biologics, especially vaccines, to counteract against this threat. In this scenario, a plant-made vaccine is a viable approach to rapidly respond to this need. The current expression technologies offer relevant paths for developing anti-COVID-19 vaccines. VLPs constitute an attractive approach for the development of efficient and safe vaccines, which is associated with high immunogenicity, preservation of the antigenic determinants, and lack of replicative capacity. Thus, VLPs based on the main SARS-CoV-2 structural proteins is an attractive approach for vaccine development against coronavirus infections. The transient expression systems based on deconstructed viral vectors and *N. benthamiana* as host will allow for immediate exploitation of plants as efficient biofactories of injectable vaccine candidates, which are expected to be implemented and entered into clinical trials in a year. The development of vaccines based on transplastomic lines or edible plant species transformed at the nuclear level and intended to result in oral vaccines (especially boosting agents to provide mucosal immunity) are considered long term goals. However, they have special importance given their low cost and potential to serve as effective boosting agents, which could lead to attractive immune profiles characterized by proper humoral response in the mucosal compartments and long-lasting protection, especially for the elderly. In parallel to these developments, the production of monoclonal antibodies in plants will provide another strategy to generate alternatives to the convalescent plasma transfusion, in which plant-made antibodies will constitute a low cost and safer intravenous treatment for critically ill patients.

Perhaps the main challenge envisioned for the development of COVID-19 plant-based vaccines will be, as is typical for all vaccines, testing their efficacy in large clinical trials to validate their safety while fulfilling the requirements of regulatory agencies. The fact that there are precedents of a plant-made biopharmaceutical approved for human use and plant-made vaccines against influenza under clinical trials (with promising safety and efficacy) is encouraging. Therefore, plant-based vaccines have a realistic potential to contribute to the fight against COVID-19.

As the COVID-19 epidemic advances, the exploitation of plant-made vaccines is a promise to generate low cost, easy to administer, and safe/effective vaccines to fight against this pandemic. The next few months will be critical to envision the real potential of this technology.

## Figures and Tables

**Table 1:** Description of the expression approaches for the production of plant-based vaccines and precedents for MERS/SARS-CoV-1 vaccines.

Approach	Attractive Features	Drawbacks	Proposed Target Antigens	MERS/SARS Precedents	Reference
Stable nuclear genome transformation	Inheritable antigen production, allows seed bank generation; post-translational modifications are performed; protocols available for several species including seed crops	Non-site specific transgene insertion; horizontal gene transfer is possible; transgene expression affected by position effects and silencing; transformation takes long time	S protein; multiepitope vaccines	The N-terminal fragment of the SARS-CoV-1 S protein (S1) was expressed in stably transformed tomato and low-nicotine tobacco plants, which induced IgA and IgG responses in mice.	[32]
Transient nuclear genome transformation	Rapid production; high productivity; implemented at the industrial level	Seed bank cannot be generated; requires purification of the antigen to eliminate toxic compounds from the host and ag-robacteria residues	S protein; multiepitope vaccines	A chimeric protein of GFP and amino acids 1-658 of the SARS-CoV-1 S protein (S1:GFP) was transiently expressed in tobacco leaves and stably transformed in tobacco and lettuce. No immunization assays were performed The SARS-CoV-1 N protein was transiently expressed in *Nicotiana benthamiana*, which induced in mice high levels of IgG1 and IgG2a and up regulation of IFN-γ and IL-10 in splenocytes. A chimeric protein of GFP and the SARS-CoV-1 S protein was transiently expressed in tobacco plants. No immunization tests were performed. The SARS-CoV-1 M and N proteins were transiently expressed in *N. benthamiana*. The N protein was antigenic but immunogenicity was not assessed.	[33,34,35]
Transplastomic technologies	High productivity; multigene expression is possible; improved biosafety since the transgene is inherited maternally; site-specific insertion through recombination; not affected by silencing or position effects	Complex post-translational modifications are not performed; transformation protocols available for few species and the generation of lines takes long time.	Multiepitope vaccines	A chimeric protein of GFP and amino acids 1-658 of the SARS-CoV-1 S (S1:GFP) was expressed in transplastomic tobacco plants.	[32,36]

**Table 2:** Vaccines against influenza based on plant-made Virus-Like Particles (VLPs), evaluated in vitro or in mice and humans.

VLPs Produced and Immunization Approach	Findings	Reference
Haemagglutinin (HA) from H1N1 A/California/07/2009 (pdmH1N1). Mice were immunized twice by the intramuscular (i.m.) route or by combining i.m. priming and (intranasal) i.n. boosting.	VLP influenza vaccination exhibited high levels of antibodies titers compared to the inactivated influenza vaccine (IIV). Lung homogenate displayed chemokine/cytokine levels and virus loads lower in the VLP groups compared to the IIV group.	[45]
HA from strains A/California/07/2009 H1N1 (H1/Cal), A/Victoria/361/11 H3N2 (H3/Vic), B/Brisbane/60/08 (B/Bris, Victoria lineage), and B/Massachusetts/02/2012 (B/Mass, Yamagata lineage). In a Phase II clinical trial, subjects were immunized with a single i.m. dose using Alhydrogel as an adjuvant.	Both homologous and heterologous antigen-specific CD4+ T cells were elicited. Additionally, production of IFN-γ, IL-2, and/or TNF-α was achieved upon ex vivo antigenic re-stimulation.	[46]
HA from A/California/07/2009 H1N1. VLPs were evaluated in vitro using human monocyte-derived macrophages.	The plant-made VLPs were efficiently captured and subjected to endosomal processing and cross-presentation.	[47]
HA from A/H1N1/California/07/09 (pdmH1N1). The inactivated H1N1 vaccine (IIV) was included as a reference vaccine. Mice were i.m.-immunized twice.	CD4+ (TNF-α, IFN-γ) and CD8+ (IFN-γ) T cell responses were higher for the plant-made vaccine than the IIV formulation. The plant-made VLP vaccine elicited stronger and more balanced immune responses than IIV.	[48]
HA from A/California/7/09 (H1N1) and A/Indonesia/5/05 (H5N1). In vitro assays were performed using mouse and human DCs. Mice were immunized by the i.m. route using Alhydrogel as an adjuvant.	Human DCs exposed to plant-made VLPs showed high stimulation in terms of secretion of IL-6, IL-10, and TNFα and CD83 expression, along with activation of CD4+ and CD8+ T cells. VLPs induced accumulation of both cDC1s and cDC2 in the draining lymph nodes of test mice. Lymphocytes from mice immunized with plant-made VLPs secreted higher concentrations of pro-inflammatory cytokines (including IL-12, IL-6, and TNF-α) upon antigenic stimulation.	[49]
HA from A/California/7/2009 or A/Indonesia/5/05 strains. In vitro assays were performed using mouse dendritic cells.	The plant-made VLPs trimmers were morphologically stable over time and interacted with activated antigen-presenting cells similar to the wild type virus.	[50]

**Table 3:** Summary of antigen design strategies rendering highly immunogenic formulations.

Approach	Attractive Features	Drawbacks	References
Epitopes fused to B subunits of cholera toxin or the heat labile enterotoxin (CTB/LTB)	Compatible with mucosal immunization	May induce tolerance	[56,57]
VLPs (native or chimeric)	Simple purification	Chimeric VLPs often have limitations on the insert size (unrelated antigen)	[58]
Immune complexes	Simple purification	High expression is required to form the complexes	[59]
ELPylated antigens	Robust expression Simple purification	Low yields when large number of ELP moieties are included	[60]
